# Cancer-associated fibroblasts in cancer drug resistance and cancer progression: a review

**DOI:** 10.1038/s41420-025-02566-x

**Published:** 2025-07-24

**Authors:** Hideyuki Masuda

**Affiliations:** Research Institute of Pharmaceutical Sciences, Faculty of Pharmacy, Musashino University, Tokyo, Japan

**Keywords:** Cancer microenvironment, Drug delivery

## Abstract

Although cancer treatment saves many lives, some types of cancer, such as pancreatic ductal adenocarcinoma (PDAC), exhibit therapeutic resistance and continue to show high mortality. Tumors in cancers such as PDAC contain a substantial amount of cancer-associated fibroblast (CAF)-secreted collagen and other extracellular matrix (ECM) components, which significantly contribute to cancer therapeutic resistance. In the tumor microenvironment, CAFs stabilize the tissue by producing ECM components, remodel ECM through degradation, induce metastasis through epithelial–mesenchymal transition, and suppress cancer immune responses. Recent advances in single-cell analysis have gradually elucidated the subtypes of CAFs and their functions, leading to the emergence of CAF-targeting therapeutic strategies. In this review, I provide an overview of CAFs, their functions and classifications, the mechanisms underlying their role in therapeutic resistance, and the current status of CAF-targeting therapeutic strategies. Moreover, I explored how we can advance cancer treatment by leveraging our understanding of CAFs.

## Facts

- Cancer-associated fibroblast (CAF) subtypes affect tumor progression, metastasis, and therapeutic resistance differently.
- Excessive extracellular matrix (ECM) in tumors limits drug penetration and T-cell infiltration.
- Combination therapy using CAF-targeting therapies can improve tumor drug penetration and T-cell infiltration.
- Understanding the characteristics of all CAF subtypes will directly help determine which CAF subtypes should be targeted for efficient cancer treatment. It will also help decide which approach—CAF depletion, CAF signal transduction inhibition, or CAF normalization—to employ for best treatment outcomes from CAF-targeting therapies.

## Open questions

- Is it possible to strategically optimize the amount of ECM in tumors to enhance drug penetration without promoting cancer metastasis?
- Given that the efficacy of CAF-targeting therapies depends on specific CAF subtypes, is it currently possible to target specific CAF subtypes to enhance treatment outcomes?
- Although there are several approaches—such as CAF depletion, CAF signal transduction inhibition, and CAF normalization—to be considered when using CAF-targeting therapies, it is not clear which one will provide the best treatment outcome.

## Introduction

Numerous medicines and therapies have been developed to treat various cancers [1]. However, given the complex nature of the tumor tissue, developing effective cancer treatments is challenging [2]. Tumors, like human organs, are composed of a variety of cells; in addition to cancerous cells, tumors contain a variety of host-derived cells and tissues, such as cancer-associated fibroblasts (CAFs), immune cells, and vascular endothelial cells [2, 3]. Thus, understanding the roles of various components within the tumor microenvironment (ECM) is crucial for developing more effective cancer treatments. CAFs act as key players due to their diverse subtypes and multifaceted functions [4, 5]. Unlike other stromal cells such as immunosuppressive cells and vascular endothelial cells, CAFs play unique roles in the development of physical barriers for drug penetration through ECM production, suppression of antitumor immunity, angiogenesis, and enhancement of cancer cell migration. These functions of CAFs contribute to therapeutic resistance and poor treatment outcomes [6–10]. In pancreatic ductal adenocarcinoma (PDAC), which is one of the most lethal cancers, the presence of abundant CAFs and CAF-produced ECM is associated with therapeutic resistance and poor treatment outcomes [11]. Therefore, investigating the characteristics of CAFs and developing CAF-targeting therapies are necessary. Recently, new cancer treatments targeting both cancer cells and CAFs have been developed and some clinical trials have been initiated [12–22].

In this review, I focused on the functions of CAFs in the development of therapeutic resistance in cancers and the progression of cancer from the viewpoint of developing new treatments. First, I overviewed the tumor microenvironment (TME) and discussed the subtypes and functions of CAF in tumor development. Finally, I summarized preclinical and clinical studies on CAF-targeting therapies.

## TME

**Fig. 1:**
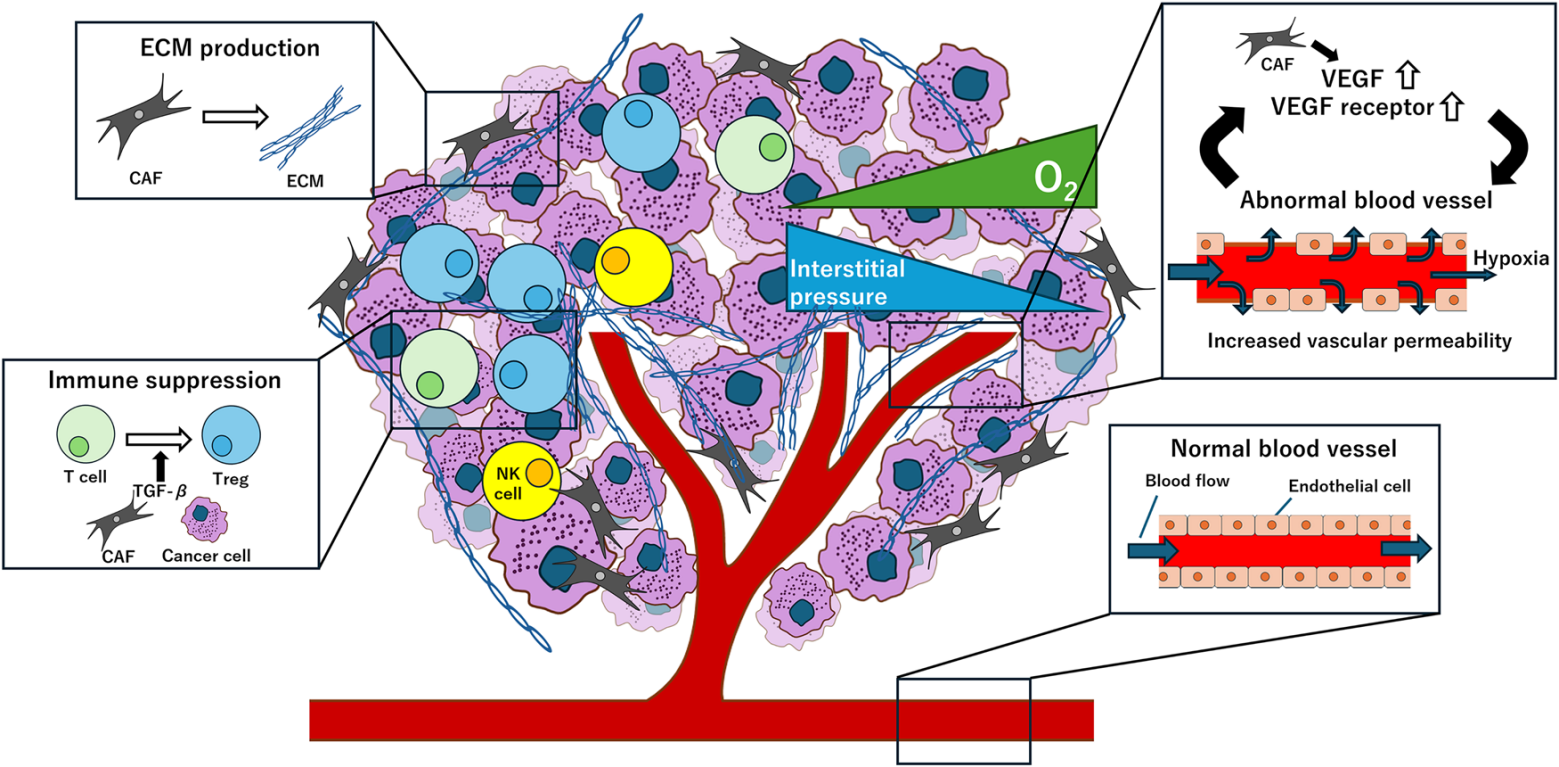
Overview of the Tumor Microenvironment (TME). Tumor tissues are not monolithic; they are composed of a variety of cell types, including cancer cells and noncancer cells. The walls of tumor vasculature are “leaky” due to being structurally incomplete compared with the selective permeable walls of the normal tissue vasculature. Consequently, the leaky vasculature prevents uniform blood supply to the tumor tissue, leading to hypoxia and increased internal pressure. Moreover, most lymphocytes within the tumor tissues differentiate into immunosuppressive cells, such as Tregs. Additionally, the fibroblasts in the TME, known as CAFs, are chronically activated and produce excessive amounts of ECM components, such as collagen.

Multiple cell types, including noncancer cells, such as immune cells, fibroblasts, and vascular endothelial cells, can be found within the tumor tissue [23] (Fig. 1). These host-derived cells play crucial roles in tumor growth. Moreover, although derived from the host, these cells differ significantly from the normal host cells in their characteristics. CAFs play a central role in the TME by interacting with and influencing the behavior of various cell types, including immune cells and vascular endothelial cells. In this section, I provide an overview of TME and CAFs without details of CAF subpopulations.

### Fibroblasts in the TME

Fibroblasts are activated into myofibroblasts in open wounds and aid in healing through collagen production [24, 25]. During this process, the activated fibroblasts express various activation markers, such as α-smooth muscle actin (SMA) and fibroblast activation protein (FAP) [4]. At the end of wound healing, the activated fibroblasts revert to resting fibroblasts. However, as suggested by the phrase “tumors: wounds that do not heal,” fibroblast activation in tumor tissue is important in cancer progression [26]. Fibroblasts in the TME are more activated than those involved in normal wound healing. In the TME, the activated fibroblasts transform into CAFs and overproduce the stromal components. Moreover, CAFs can differentiate from stellate cells, endothelial cells, pericytes, adipocytes, and mesothelial cells through the action of inflammatory cytokines, local hypoxia, and CAF-derived exosome in the TME [27–31]. During early stage in tumor progression, secreted cytokines from cancer cells, such as IL-1β, guide normal fibroblasts to differentiate into proinflammatory CAFs depending on NF-κB activation [32]. Similarly, hypoxia conditions induce the differentiation of pancreatic stellate cells cocultured with tumor organoids into CAFs expressing an inflammatory phenotype [33]. miRNA contained in CAF exosome can also transform normal fibroblasts into CAFs [34]. These transformations are irreversible due to epigenetic changes that make the reversal of CAFs into resting fibroblasts unlikely [35]. These unique properties of CAFs are closely related to their origin and the activation process. CAFs are presumed to possess heterogeneous properties due to their abnormal activation in the TME and diverse cellular origins (beyond fibroblasts).

### Immune cells in the TME

Various immune cell types, such as T cells, macrophages, and natural killer (NK) cells, are found in the TME. These immune cells exhibit potential antitumor immunity [36]. Antitumor immunity typically involves the immune responses to tumor neoantigens [37, 38]. CAFs play a significant role in modulating the immune landscape to promote tumor growth. For example, CAFs induce the differentiation of T cells into regulatory T cells (Tregs) via antigen presentation without costimulatory molecules [39]. In addition, the migration of M2 macrophages and myeloid-derived suppressor cells (MDSCs) is induced by the secretion of cytokines and chemokines by CAFs [32]. These mechanisms contribute to drug resistance and poor prognosis, particularly for cancer immunotherapy [40].

### Blood vessels in the TME

Cancer cells proliferating uncontrollably require blood supply to grow. Then, vascular endothelial growth factor (VEGF), which induces angiogenesis, is frequently induced in the TME [41]. CAFs also induce angiogenesis via the secretion of VEGF and the VEGF-independent STAT3 signaling pathway [4, 34, 42]. However, the tumor vasculatures have a discontinuous vascular endothelium compared with normal tissue vasculature [43]. As a result, the entire TME experiences high interstitial pressure and hypoxia, which support both the tumor growth and the development of therapeutic resistance [44, 45].

### Extracellular matrix (ECM) in the TME

ECM is the noncellular constituent found in all organs and tissues. The major ECM proteins include collagen, proteoglycan, and elastin [46]. In the TME, the ECM undergoes dynamic remodeling due to its continuous generation and degradation [47]. As previously stated, CAFs contribute to ECM production and degradation by secreting matrix metalloproteinases (MMPs) [48–50]. The ECM not only mechanically supports cancer tissues but also acts as a reservoir for growth factors, releasing them to cancer cells under specific conditions. It also acts as a scaffold that promotes cancer cell interactions with growth factors. Furthermore, ECM remodeling (via degradation) modulates the interaction of scaffold with cancer cells and as well as regulated cellular metabolism and other signaling pathways [51]. Details regarding ECM functions have been discussed in the section titled “Mechanisms of CAF-mediated tumor progression and therapeutic resistance.”

## Classification of CAFs and their role in tumor growth

**Fig. 2:**
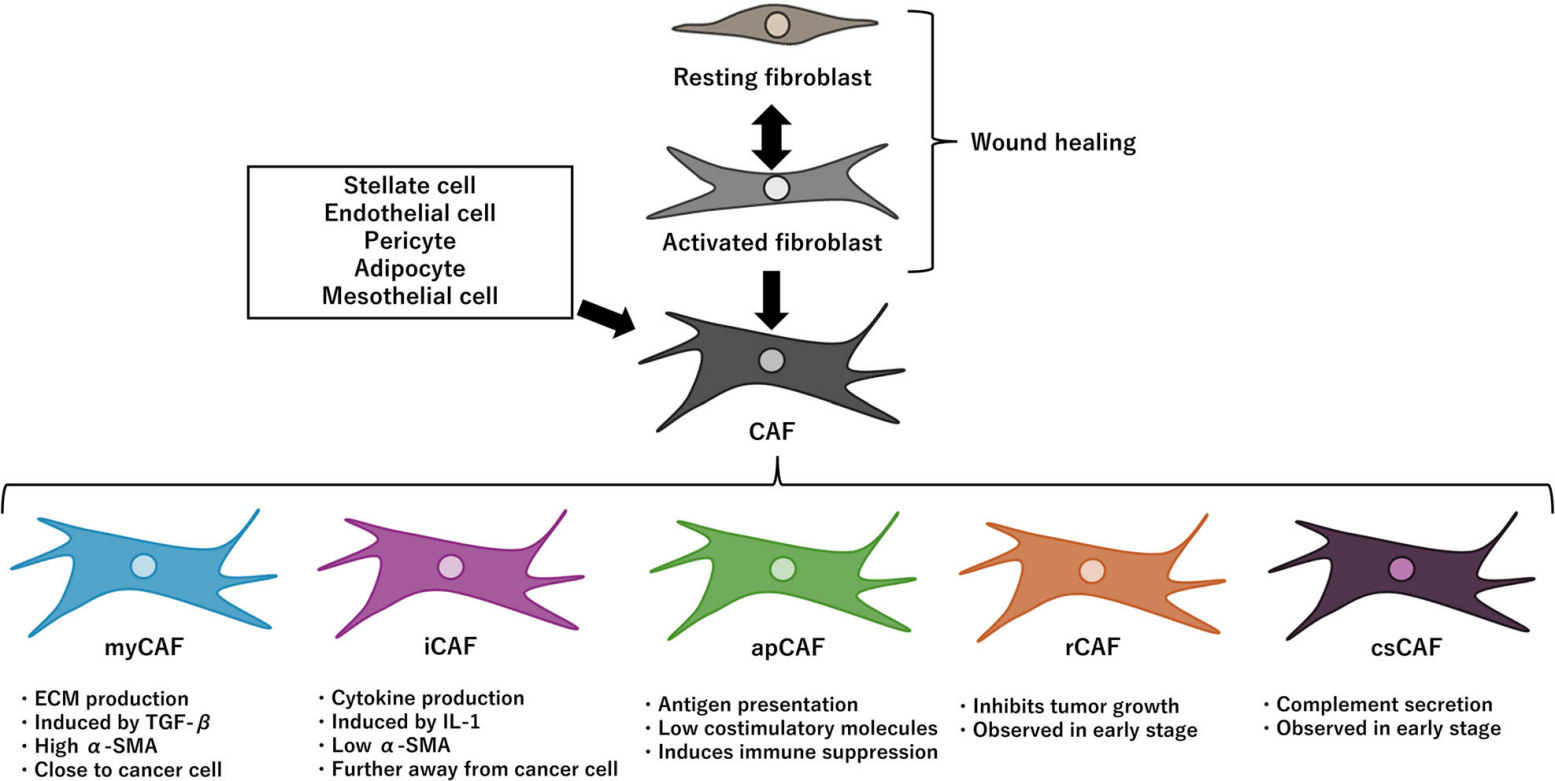
Overview of CAFs. Fibroblasts, which are primarily responsible for the origin of CAFs, are activated during wound healing and produce ECM. Once the wound heals, these fibroblasts return to a quiescent state. However, cancer-associated fibroblasts (CAFs) remain perpetually activated in tumors. The origin of CAFs is not limited to fibroblasts from the primary tumor; they also originate from BM-MSCs, pancreatic stellate cells, etc. CAFs are not a homogeneous cell population but rather a heterogeneous group of several subtypes. Each CAF subtype has different functions, ranging from producing ECM and cytokines, suppressing cancer immune responses via presenting antigens (in the absence of costimulatory molecules), and even inhibiting tumor growth.

Single-cell analysis has revealed that CAFs are not a homogeneous cell population [52]. Although the primary function of CAFs is to produce ECM components, they are involved in various other functions [4, 35]. These functions both assist and suppress tumor growth [4, 35]. In this section, I discuss various CAF subtypes and their role in tumor growth (Fig. 2).

### Myofibroblastic CAFs (myCAFs)

myCAFs play a significant role in ECM production [4]. myCAFs express high levels of α-SMA, a marker of the myofibroblast phenotype, and have myofibroblast characteristics that play a key role in wound healing [53]. Therefore, myCAFs primarily contribute to ECM production [48, 49]. myCAFs are located near tumor cells and are highly responsive to TGF-β [48, 53, 54]. In fact, TGF-β promotes the differentiation of progenitor cells into myCAFs, leading to the excessive production of ECM [55]. The spatial proximity of myCAFs and cancer cells is hypothesized to effectively promote treatment resistance through the physical barrier formed by the ECM. Clinically, a high ratio of myCAFs in tumor tissue is related to abundant ECM and worse prognosis [53, 56]. Contrarily, myCAFs may partly inhibit tumor growth. For instance, myofibroblast depletion leads to aggressive mouse pancreatic cancer and metastatic colorectal cancer [57, 58]. Furthermore, type 1 collagen deletion in myCAFs enhances tumor progression and suppresses cancer immunity via the migration of MDSCs [59]. Thus, myCAFs play roles in both promoting and inhibiting tumor growth.

### Inflammatory CAFs (iCAFs)

iCAFs produce inflammatory cytokines, such as CXCL-1, IL-1, IL-6, stromal cell-derived factor-1, hepatocyte growth factor, and CCL17, via the JAK/STAT and NF-kB signaling pathways [60, 61]. Cytokines secreted by iCAFs induce the migration and activation of MDSCs, M2 macrophages, and Tregs within tumors, thereby suppressing the cancer immune response and contributing to cancer growth [61, 62]. iCAFs, which exhibit high expression of the IL-1 receptor and low expression of α-SMA, are induced by IL-1 and are located distant from cancer cells [55]. Moreover, iCAFs and myCAFs have a reciprocal relationship, converting into one another in response to cytokines, such as IL-1 and TGF-β [48]. Although the functional implications of the spatial relationship between iCAFs and cancer cells remain unclear, this arrangement may confer advantages to tumor growth. In particular, the positioning of iCAFs outside the physical barrier of ECM constructed by myCAFs allows efficient secretion of cytokines, potentially promoting the recruitment of Tregs and other immunosuppressive cell types.

### Antigen-presenting CAFs (apCAFs)

apCAFs are a subpopulation of CAFs characterized by the expression of MHC class II and CD74. However, apCAFs cannot activate immune functions like other antigen-presenting cells because they show low expression of additional costimulatory molecules, such as CD80, CD86, and CD40 [39]. apCAFs directly ligate and induce naive CD4^+^ T cells to become Tregs in an antigen-specific manner [31]. apCAFs are especially found in PDAC; and these apCAFs are differentiated from mesothelial cells through downregulation of its mesothelial features and gaining of fibroblastic features. This differentiation is mediated by IL-1 and TGF-β [31]. Regarding the spatial distribution of apCAFs in the TME, it has been suggested that in the breast cancer TME, apCAFs may not exhibit specific spatial distribution characteristics as iCAFs or myCAFs [63].

In addition, apCAFs that activate CD4^+^ T cells have been recently discovered. In human lung tumor tissues, apCAFs surrounded by CD4^+^ T cells, not Tregs, were observed [64]. These apCAFs activate CD4^+^ T cells by presenting antigens on their MHC class II molecules [64]. This fact indicates the possibility that CAFs exhibit functional diversity across different cancer types, even within the same molecular subtype.

### Cancer-restraining CAFs (rCAFs)

CAFs contain subtypes that inhibit tumor development [35]. rCAFs, unlike the previously mentioned types, are thought to suppress tumor growth [65]. rCAFs are a subset of CAFs that are observed predominantly in the early stages of tumor tissue; these CAFs decrease as the tumor tissue grows [65]. No reports on the spatial distribution of rCAFs are available. This lack of information is likely due to the difficulty in identifying rCAFs with increasing growth of the tumor tissue. Originally, it was discovered that normal fibroblasts play a role in inhibiting cancer cell growth [66, 67]. rCAFs with properties similar to normal fibroblasts possess unique markers. Meflin, a marker of normal fibroblasts, inhibits lysyl oxidase, which induces collagen crosslinking, thereby reducing the collagen content in the ECM and improving the softening of the tumor tissue and drug penetration [68]. Patients with pancreatic cancer expressing high levels of meflin showed significantly longer postoperative survival rates than those expressing low levels of meflin [69, 70]. Asporin, a marker of normal fibroblasts, inhibits the TGF-β/Smad signaling pathway by binding to TGF-β1 and is derived from fibroblasts [71]. Patients with breast cancer expressing high levels of asporin are associated with a better prognosis than those with low asporin expression [72]. Contrarily, prostate tumor allografts in asporin-null mice showed a decrease in the number of cancer stem cells and tumor-associated mesenchymal stromal cells and an increase in the number of CD8^+^ cells [73]. These two conflicting reports regarding asporin may rely on the cancer type and the condition of the tumor microenvironment and should therefore be interpreted with caution. Understanding and appropriately utilizing rCAFs are crucial for improving the efficiency of CAF-targeting therapies.

### Complement-secreting CAFs (csCAFs)

Chen et al. identified a new subtype of CAFs present only in early stage PDACs. csCAFs mainly secrete complement proteins, such as C3 and C7 [74]. The specific functions and spatial distribution of csCAFs have not yet been elucidated; however, in neoadjuvant therapy for PDAC, the expression of complement proteins is enhanced by secretion from CAFs and is associated with improved patient survival [75]. This indicates that csCAFs or the secretion of complement proteins from CAFs may affect cancer treatment.

## Mechanisms of CAF-mediated tumor progression and therapeutic resistance

**Fig. 3:**
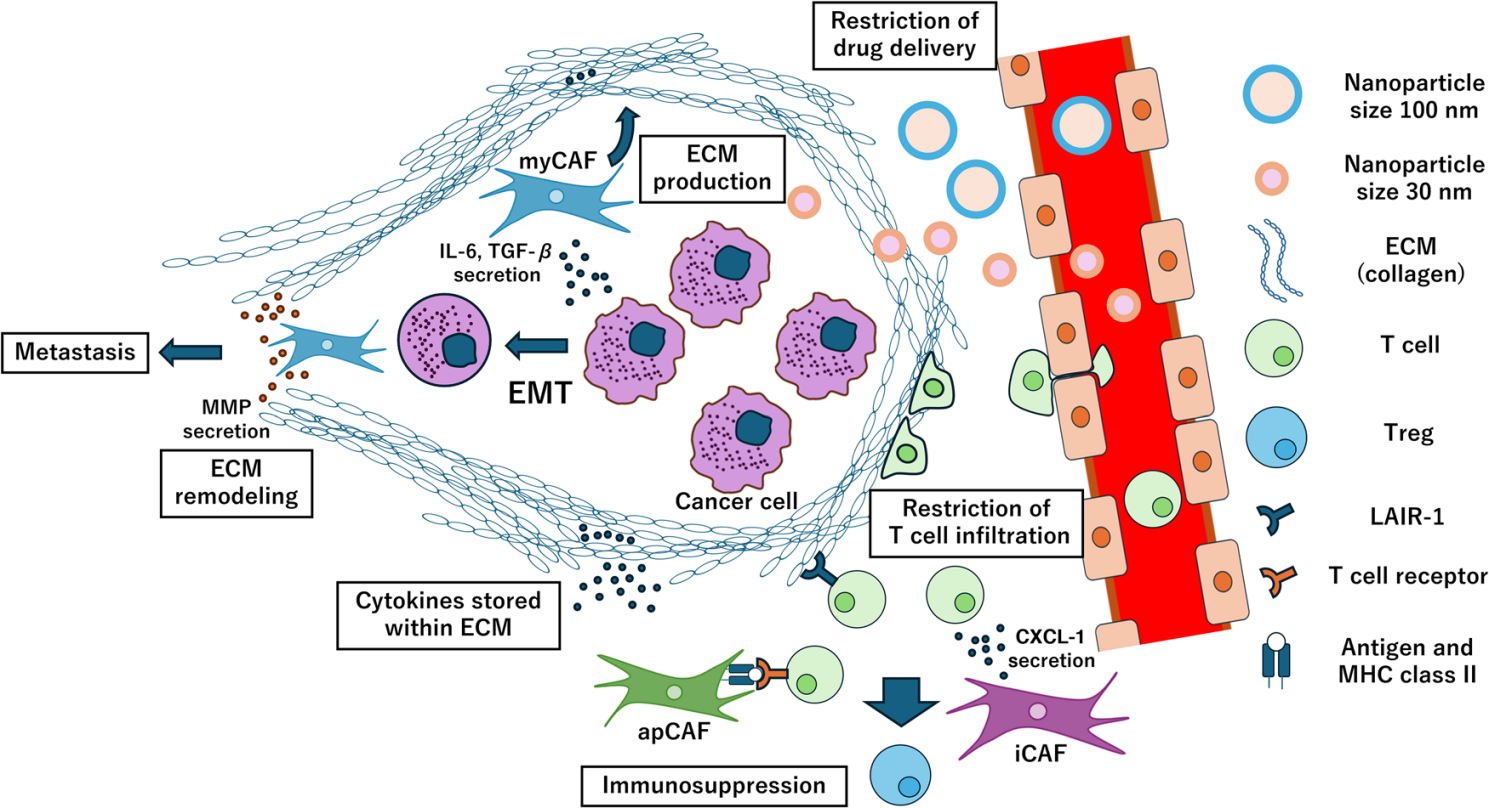
Therapeutic resistance induced in cancers by excessive ECM in the TME. The mechanisms by which CAFs induce therapeutic resistance in cancer cells can be broadly classified into three categories: (i) hindering of drug penetration and T-cell infiltration: the ECM produced by myCAFs is very dense and hinders drug penetration and T-cell infiltration. It is hypothesized that drugs can penetrate the tissue across blood vessels through the enhanced permeability and retention (EPR) effect, facilitated by the “leaky” tumor vasculature walls. However, the excessively produced ECM creates a barrier that prevents these particles from penetrating deep into the tumor tissue. For example, while nanoparticles of ~100 nm in diameter can traverse the tumor vasculature walls, the dense ECM obstructs their further movement into the tumor core. Meanwhile, smaller nanoparticles (30 nm diameter) are delivered into the tumor interior without being impeded by the high-density ECM. Similarly, T cells are unable to traverse the high-density ECM using ameboid movement to reach the tumor interior. (ii) Suppression of anticancer immunity: inflammatory and antigen-presenting CAFs (iCAFs and apCAFs) and ECM collagen suppress anticancer immunity by inducing the formation of Tregs. iCAFs primarily secrete CXCL-1, which promotes the migration of T cells. apCAFs also induce Tregs via antigen presentation (in the absence of costimulator molecule). Collagen induces immunosuppression through interactions mediated by LAIR-1. (iii) Induction of EMT: CAFs induce EMT and cancer metastasis by secreting cytokines that remodel the ECM. “Remodeled ECM tracks” are formed by cytokine-induced matrix metalloproteinases (MMPs), along which the cancer cells move. With ECM remodeling, cytokines stored within the ECM are released.

**Fig. 4:**
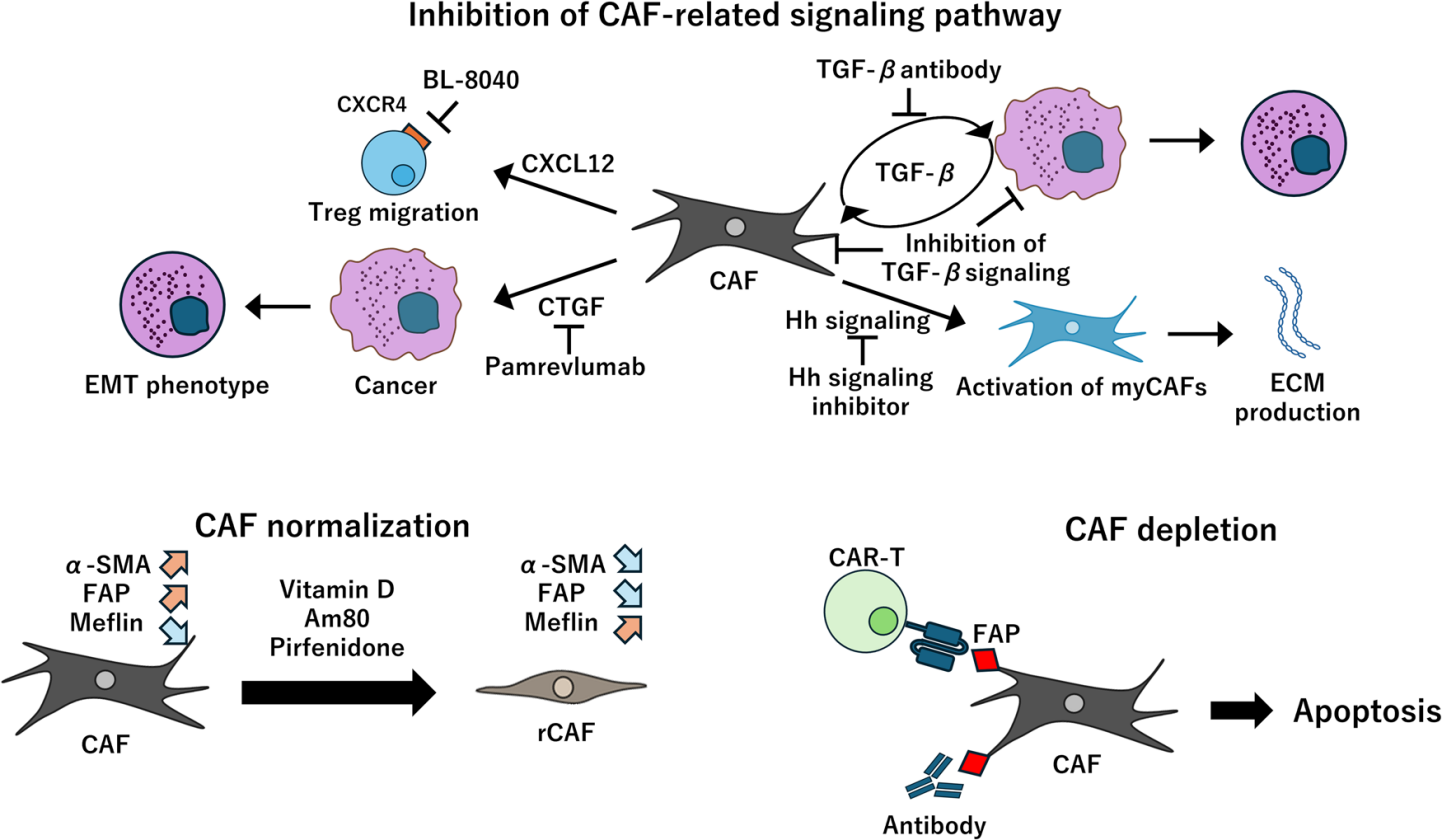
Overview of the approaches used in CAF-targeting therapies. Therapies targeting CAFs broadly use three approaches. The first approach involves inhibiting CAF activation or the cytokines secreted by CAFs. This method primarily targets the signaling pathways. The second approach focuses on the elimination of CAFs. CAR-T cells targeting FAP or the use of antibodies against FAP aim to remove CAFs, thereby reducing the amount of ECM and potentially improving treatment sensitivity. The third approach seeks to normalize CAFs. Agents like pirfenidone and various vitamins are used to differentiate activated CAFs into rCAFs, thereby improving the TME.

As discussed in the previous sections, CAFs produce ECM components like collagen and have various functions, including cytokine production, antitumor immune response suppression, and tumor growth inhibition. In this section, the contribution of CAFs to cancer growth, metastasis, and resistance to treatment will be elucidated (Figs. 3 and 4).

### ECM production in tumor progression and therapeutic resistance

One of the primary roles of fibroblasts is to produce ECM when activated. While fibroblasts are typically activated in response to wounds, CAFs remain in a constitutively activated state [4, 25, 76]. The excessive production of ECM poses several challenges in cancer therapy.

First, it inhibits drug penetration by diminishing the enhanced permeability and retention (EPR) effect. In cancer treatments, the EPR effect occurring due to the “leaky” vasculature and the lack of lymphatic drainage is expected to facilitate tumor drug penetration [26]. However, this effect may be limited in various cancer types [77, 78], especially in PDAC and breast cancer, which are characterized by blood vessels embedded within abundant ECM [79, 80]. For instance, in clinical trials, compared with conventional chemotherapy, the liposome-encapsulated doxorubicin formulations designed to leverage the EPR effect could reduce some adverse events in metastatic breast cancer but could not significantly improve the progression-free survival [81]. Similarly, a meta-analysis of clinical trials comparing the efficacy of liposomal chemotherapy with that of conventional chemotherapy confirmed that there was no significant difference in efficacy between the two treatment methods [82]. Lack of improvement in treatment efficacy when using liposome-encapsulated drugs can be attributed to the diameter of the encapsulating liposome. While the “leaky” tumor vasculature can allow nanoparticles as large as 100 nm in diameter to pass, the particle diameter must be ≤30 nm for the effective delivery of nanoparticles to the core of tumors having abundant ECM [83–85]. However, the particle size of general liposomal formulations is around 100 nm in diameter. Thus, the absence of improved efficacy in treatments involving liposome-encapsulated drugs could be due to the inability of liposomal formulations to infiltrate the cancer tissue after passing through the tumor vasculature [86]. Therefore, excessive/abundant ECM probably negates the EPR effect and limits drug penetration into tumor tissues.

Second, like in the case of drug penetration, high interstitial pressure and the overexpressed ECM restrict the infiltration of T cells into the tumor tissue. High interstitial pressure inhibits T cells from extravasating from blood vessels and infiltrating cancerous tissues [87]. A loosely aligned ECM scaffold guides the ameboid movement of T cells, which does not rely on adhesion molecules [88, 89]. However, the overexpressed ECM scaffold has a very fine lattice structure, which impedes the amoeboid T-cell movement as the physical barrier [90]. Thus, high density network of ECM reduces physical spaces and inhibits the migration of T cells into tumor tissue. This cell–cell contact plays a pivotal role in the activation and execution of antitumor immune responses, ultimately influencing treatment outcomes [91]. Therefore, the inhibition of T-cell infiltration through ECM is one of the risk factors of therapeutic resistance in cancer immunotherapy [92].

Third, specific domains of the ECM scaffold can bind and activate receptors on the cell surface [93]. For example, the T cells are suppressed through their interactions with the ECM scaffold. Immune cells, including T cells and NK cells, express a receptor called leukocyte-associated Ig-like receptor 1 (LAIR-1) [94]. LAIR-1 binding to the collagen domain activates inhibitory signaling pathways, leading to the suppression of T cells and NK cells. This interaction may hinder anticancer immunity and lead to the survival of cancer tissues [95, 96]. The interaction between fibronectin and leukocyte immunoglobulin-like receptor B4 (LILRB4) has similar effects [97, 98].

Fourth, the ECM acts as a reservoir of growth factors. For instance, pro-TGF-β is synthesized with its N-terminal precursor (also known as latency-associated peptide [LAP]). LAP and mature TGF-β remain attached via noncovalent bonds, forming a latent complex. This LAP–TGF-β complex can further bind to the large latent TGF-β-binding protein (LTBP), which in turn interacts with fibrillins and fibronectin. Thus, LAP shields mature TGF-β from proteolytic degradation, allowing it to be stored within the ECM. Certain situations like ECM degradation in the TME releases TGF-β, allowing it to exert its effects on the surrounding cells [93, 99, 100]. In addition, the mechanical forces of ECM affect cancer cells and CAFs. For example, the elastic modulus of the normal breast is ~1000 Pa. Further, in breast cancer tissue, the elastic modulus can increase to a maximum of 4000 Pa [101, 102]. ECM stiffness promotes stemness in cancer cells, while excessive stiffness paradoxically induces cancer cell dormancy. These findings have significant implications for cancer metastasis and recurrence [103]. Moreover, in colorectal cancer liver metastases, hepatic stellate cells induce the secretion of free fatty acids (FFAs) through the mechanical forces of ECM, and cancer cells gain therapeutic resistance via FFA intake and oxidation [104]. Therefore, ECM acts as a physical barrier and ligand, stores growth factors, and controls factors through mechanical forces to develop tumor tissue.

### Induction of cancer cell migration

CAFs are involved in the distant metastasis of cancer cells. Primarily, there are two main mechanisms through which metastasis is induced: (i) individual cell invasion and (ii) ECM remodeling. In individual cell invasion, metastasis is promoted by the induction of epithelial–mesenchymal transition (EMT) of cancer cells [105, 106]. EMT is a cellular process through which epithelial cells acquire mesenchymal phenotypes and behavior [107]. CAFs play a crucial role in the induction of EMT [105]. Several cytokines, such as TNF-α, IL-6, IL-1β and TGF-β, are involved in the EMT of cancer cells [108–113]. These cytokines are mainly secreted by tumor‐associated macrophages, MDSCs, and T helper cells/cytotoxic T cells [114]. In addition, IL-6, secreted in a paracrine manner by iCAFs, can induce EMT in human bladder cancer cells, promoting enhanced proliferation, migration, and invasion [111]. Although studies directly investigating the relationship between iCAFs and cancer EMT are few, the potential for CAFs to induce EMT is considered significant, given that iCAFs secrete cytokines causing EMT [105, 115].

In the ECM remodeling mechanism, the tumor ECM is remodeled by MMPs secreted by CAFs. Cancer cells infiltrate through tracks formed by the remodeled ECM [116]. An in vitro experiment involving coculturing of CAFs and cancer cells within a type I collagen gel revealed a migration pattern wherein CAFs infiltrated the gel first, followed by the subsequent infiltration of cancer cells [117]. Moreover, cancer stem cells are involved in metastasis. In the above experimental system, both cancer stem cells and noncancer stem cells infiltrated to similar extents when cultured in the absence of CAFs. However, when cocultured with CAFs, cancer stem cells showed significantly enhanced infiltration compared with noncancer stem cells [118]. As previously stated, the degradation of the ECM by MMPs can reduce the stiffness of the tumor tissue. This decrease in mechanical resistance is suggested to facilitate cancer cell migration [103, 119, 120]. These findings suggest the potential contribution of CAFs to the metastatic process.

## CAF-targeting therapies

As discussed in the previous sections, CAFs are involved in cancer progression, metastasis, and drug resistance, potentially leading to poor prognosis. Consequently, recent research efforts have focused on developing CAF-targeting therapies. This section provides an overview of the existing CAF-targeting therapies (Fig. 4). Details of the clinical trials on CAF-targeting therapies are summarized in Table 1. The trials included here (listed on clinicaltrials.gov) include those recruiting patients from January 1, 2014 to October 31, 2024.

**Table 1:** Clinical trials of CAF-targeting therapies.

Trial	Cancer types	CAF target molecule	Interventions	Approach	Clinical trial phase
NCT05653284	Malignant Tumor	TGF-β	AK130 (Anti-TIGIT antibody/Anti-TGF-β-receptor II bifunctional fusion protein)	Signaling inhibition	1
NCT05717348	Solid Tumor		ES014 (Anti-CD39/Anti-TGF-β bispecific antibody)		1
NCT06543056	Solid Tumor				2
NCT02581787	Non-Small Cell Lung Cancer		Fresolimumab (Anti-TGF-β monoclonal antibody)		1/2
NCT03206177	Ovarian Cancer		Galunisertib (TGF-β receptor 1 inhibitor)		1
NCT02734160	Metastatic Pancreatic Cancer				1
NCT05386888	Non-Small Cell Lung Cancer		GFH018 (TGF-β receptor 1 inhibitor)		2
NCT06223308	Cervical Cancer		HB0028 (Anti-PD-L1/anti-TGF-β bifunctional fusion protein)		1/2
NCT05304936	Pancreatic Cancer		HCW9218 (TGF-β antagonist/IL-15 protein complex)		1/2
NCT05322408	Solid Tumor				1
NCT05821595	Solid Tumor		JYB1907 (Anti-TGF-β1 monoclonal antibody)		1
NCT02178358	Hepatocellular Carcinoma		LY2157299 (TGF-β receptor 1 inhibitor)		2
NCT02452008	Prostate Cancer				2
NCT02937272	Solid Tumor		LY3200882 (TGF-β receptor 1 inhibitor)		1
NCT04481256	Esophageal Cancer		M7824 (Anti-PD-L1/Anti-TGF-β bifunctional fusion protein)		Not Applicable
NCT04432597	HPV-Positive Cancer				1/2
NCT04574583	Solid Tumor				1/2
NCT03451773	Pancreatic Cancer				1/2
NCT04296942	Breast Cancer				1
NCT04501094	Urothelial Cancer				2
NCT04247282	Head and Neck Cancer				1/2
NCT04246489	Uterine Cervical Neoplasms				2
NCT03620201	Breast Cancer				1
NCT04428047	Head and Neck Cancer				2
NCT05005429	Mesothelioma				2
NCT05445882	Castration-Resistant Prostate Cancer				2
NCT02947165	Breast Cancer/Lung Cancer/ Hepatocellular Cancer/ Colorectal Cancer/Pancreatic Cancer/ Renal Cancer		NIS793 (Anti-TGF-β1 monoclonal antibody)		1
NCT04952753	Metastatic Colorectal Cancer		NIS793 (Anti-TGF-β1 monoclonal antibody)		2
NCT06079346	Pancreatic Cancer		OT-101 (TGF-β2 antisense oligodeoxynucleotide)		2/3
NCT05935774	Non-Small Cell Lung Cancer				2
NCT05537051	Solid Tumor		PM1021 (Anti-PD-L1/Anti-TGF-β bifunctional fusion protein)		1
NCT04954456	Advanced Malignant Tumor		QLS31901 (Anti-PD-L1/Anti-TGF-β bifunctional fusion protein)		1
NCT04729725	Malignant Solid Neoplasm		SAR-439459 (Anti-TGF-β monoclonal antibody)		1
NCT04423380	Solid Tumor		SH3051 (TGF-β receptor 1 inhibitor)		1
NCT04560244	Non-Small Cell Lung Cancer		SHR-1701 (Anti-PD-L1/Anti-TGF-β bifunctional fusion protein)		2
NCT05149807	Gastric Cancer				2/3
NCT04844983	Squamous Cell Carcinoma In Situ		STP705 (TGF-β1 and COX-2 siRNA)		2
NCT04862767	Solid Tumor		TASO-001 (TGF-β2 targeting antisense oligonucleotide)		1
NCT04064190	Urothelial Carcinoma		TEW-7197 (TGF-β receptor 1 inhibitor)		2
NCT02160106	Advanced-Stage Solid Tumors				1
NCT05198531	Metastatic Nasopharyngeal Cancer		TQB2868 (Anti-PD-L1/Anti-TGF-β bifunctional fusion protein)		1/2
NCT05121363	Endometrial Carcinoma				2
NCT05198505	Malignant Tumor				1
NCT05068921	Cervical Cancer				1
NCT05154630	Soft-Tissue Sarcoma				1/2
NCT05262101	High-Grade Sarcoma				2
NCT04958434	HPV-Positive Cancer		TST005 (Anti-PD-L1/Anti-TGF-β bifunctional fusion protein)		1
NCT03143985	Multiple Myeloma		Vactosertib (TGF-β receptor 1 inhibitor)		1
NCT05400122	Colorectal Cancer				1
NCT06044311	Esophageal Cancer				2
NCT05028556	Solid Tumor		Y101D (Anti-PD-L1/Anti-TGF-β bispecific antibody)		1
NCT05228600	Solid Tumor		YL-13027 (TGF-β receptor 1 inhibitor)		1
NCT01601184	Medulloblastoma	Sonic Hedgehog Pathway	Vismodegib (Hedgehog inhibitor)		1/2
NCT02907099	Pancreatic Cancer	CXCR4	BL-8040 (CXCR4 antagonist)		2
NCT01010880	Multiple Myeloma		BKT140 (CXCR4 antagonist)		1/2
NCT01359657	Multiple Myeloma		BMS-936564 (CXCR4 antagonist)		1
NCT02737072	Solid Tumor		LY2510924 (CXCR4 antagonist)		1
NCT03932565	Solid Tumor	FAP	CAR-T cell (Nectin4/FAP-targeted CAR-T cells)	Depletion	1
NCT05547321	Solid Tumor		OMTX705 (Anti-FAP-antibody–drug conjugate)		1
NCT04467723	NSCLC Stage IV | NSCLC, Recurrent	TGF-β1, 2	Pirfenidone	Normalization	1/2
NCT06484153	Colorectal Cancer				1/2
NCT03177291	Non-Small Cell Lung Cancer				1
NCT05064618	Pancreatic Cancer	Meflin	Am80 (Tamibarotene)		1/2

### Cancer treatment strategies that rely on inhibiting the CAF-related signaling pathways

In the TME, cancer cells induce CAFs to act in ways that favor their own progression through cytokine secretion. Therefore, inhibiting this phenomenon could prevent the CAF-mediated growth of tumor tissue. As mentioned earlier, TGF-β is involved in the activation and differentiation of various CAFs, such as myCAFs [48, 53, 54, 121]. Moreover, TGF-β is also secreted by CAFs and induces EMT in cancer cells. Therefore, inhibiting TGF-β is expected to be effective against both cancer cells and CAFs. In fact, over the past decade, numerous clinical trials have been actively conducted on novel therapies targeting TGF-β in combination with immune checkpoint inhibitors. However, in a mouse study, inhibiting TGF-β contributed to pancreatic cancer progression [122]. Moreover, in a clinical trial, inhibiting TGF-β signaling was not effective, as observed in preclinical trials; moreover, it caused side effects [12]. Additionally, inhibiting TGF-β after EMT in cancers might potentially enhance EMT [12]. This necessitates careful consideration of the timing of TGF-β inhibition with respect to the cancer stage. Other factors such as connective tissue growth factors (CTGFs) secreted by CAFs in the TME contribute to tumor progression by regulating proliferation and EMT; high levels of CTGFs are correlated with poor prognosis [123–125]. Thus, inhibiting CTGFs could suppress the growth of fibroblast-activated cancer cells and may therefore serve as an effective strategy in cancer treatment [126]. In a clinical study, the combination therapy of gemcitabine/nab-paclitaxel with the anti-CTGF-1 antibody pamrevlumab enhanced treatment responsiveness in patients with pancreatic cancer without increasing the toxicity [127].

In a different strategy, inhibiting the CXCR4-CXCL12 pathway (which is activated by FAP^+^ CAFs) could be effective in treating cancers. The activation of the CXCR4-CXCL12 pathway enhances the migration of Tregs; this migration is correlated with poor prognosis in patients with breast cancer [128–131]. In a clinical trial, BL-8040, a CXCR4 antagonist, increased CD8^+^ T-cell infiltration and decreased Treg infiltration in patients with PDAC [22]. The deficiency of sonic hedgehog (Shh)—which is one of the hedgehog (Hh) family members and a soluble ligand overexpressed by cancer cells in PDAC that induces fibroblast-rich TME—induces tumor growth. Shh-deficient tumors lack stroma and are aggressive [132]. Similarly, inhibiting the Hh signaling pathway alters the subtype composition of CAF. Hh signaling is more activated in myCAFs than in iCAFs. Hh signaling pathway inhibition using a smoothened antagonist decreased the number of tumor-restraining myCAFs and increased the number of tumor-promoting iCAFs. Additionally, decreased CD8 + T cells and increased Tregs in the TME were observed. The authors concluded that an increase in iCAFs induced the TME state in which cancer immune responses are suppressed because of cytokine secretion, resulting in decreased CD8^+^ T cells and increased Tregs [16]. Contrarily, inhibiting Hh signaling enhanced the normalization of blood vessels and the penetration of gemcitabine via reduction of ECM [17]. However, in a clinical trial, a combination therapy using gemcitabine and a Hh signaling inhibitor did not improve progression-free survival [18].

In conclusion, in addition to presenting promising opportunities, targeting CAF-related signaling pathways poses significant challenges in cancer treatment. While the strategies involving inhibition of pathways such as TGF-β, CTGF, CXCR4-CXCL12, and hedgehog have shown potential in preclinical studies, their clinical efficacy results have been mixed. The complex interplay between these pathways and the TME as well as the timing of interventions appear to be critical factors influencing treatment outcomes. Future research should focus on developing strategies that can selectively modulate CAF functions while minimizing unintended consequences on tumor behavior and immune responses.

### Cancer treatment strategies that rely on depleting CAFs

The key idea of depleting CAFs to treat cancers has been tried in several preclinical and clinical studies. Chimeric antigen receptor (CAR)-T-cell therapy has been used strategically to deplete CAFs. CAR-T cell therapy utilizes genetically engineered T cells to target specific antigens and is currently used in acute lymphoblastic leukemia treatment [133]. Experiments targeting FAP with CAR-T cell therapy have been reported. FAP-targeting CAR-T cell therapy reduces the amount of collagen in the TME and improves the infiltration of T cells that were previously inhibited by physical barriers [13, 14, 134, 135]. Therefore, FAP-targeting CAR-T cell therapy is expected to show higher efficacy when combined with other cancer immunotherapies [136]. In a different report, an oncolytic virus targeting CD3 and FAP induced an increase in intratumoral T-cell accumulation and a decrease in FAP^+^ cells [15]. In a mouse model with a humanized immune system, OMTX705, an anti-FAP mAb that is conjugated to cytolysin, increased CD8^+^ T-cell infiltration and decreased α-SMA^+^ CAFs [137]. However, because tumor tissue shrinkage was observed on the third day after administration and CAF reduction was observed on the 24th day, the mechanism of the antitumor effect of this treatment requires careful interpretation.

However, in some cases, the results were not as expected. For instance, specific depletion of myofibroblasts using compound genetic mouse models of PDAC led to invasive tumor cells and reduced survival rates [57]. The same study also reported that the amount of type 1 collagen in the TME was reduced. Based solely on that result, it is expected that the reduction in type 1 collagen enhances tumor drug penetration. However, they reported that PDAC did not show improvement with gemcitabine administration, instead their results indicated a worsening condition, such as increase in the number of Tregs and enhancement of EMT. Thus, the authors inferred that myofibroblasts protect against tumor growth.

Overall, CAF depletion strategies, particularly those targeting FAP^+^ CAFs using CAR-T cell therapy, have shown promise in preclinical studies by reducing collagen content and improving T-cell infiltration in the TME. However, this approach presents complex challenges, as evidenced by studies on myofibroblast depletion in PDAC models. These conflicting results highlight the dual nature of CAFs in tumor progression and underscore the need for careful consideration of targeting specific CAF subtypes in cancer treatment strategies. However, specific markers that clearly distinguish between the tumor-promoting and tumor-suppressing CAF subtypes are yet to be identified. Therefore, selective depletion of CAFs that contribute to tumor growth is very challenging.

### Cancer treatment strategies that rely on normalizing CAFs

While fibroblasts in normal tissue are only activated during wound healing [25], those in the tumor tissue are constitutively activated. Therefore, quiescing CAFs that continue to produce excessive ECM is considered an effective approach to improve TME. Pirfenidone—an antifibrotic drug used for the treatment of idiopathic pulmonary fibrosis—regulates pancreatic stellate cells and inhibits the overexpression of collagen type I and EMT via reducing TGF-β1 and 2 [19, 138, 139]. In an non-small-cell lung cancer mouse model, combination therapy with pirfenidone and cisplatin induced apoptosis in CAFs and reduced TGF-β1 [20]. In addition, all-trans retinoic acid and vitamin D normalize CAFs and reduce the ECM surrounding the pancreatic cancer cells [140, 141]. These changes induce the migration of tumor-suppressive immune cells into the tumor. Moreover, the reduction of ECM is expected to enhance drug penetration and improve chemotherapy efficacy.

Recently, meflin has been identified as a marker of CAFs that inhibit PDAC tumor growth. Meflin inhibits collagen linking via inhibiting lysyl oxidase and delays the progression of drug-induced pulmonary fibrosis [68, 142]. Am80, one of the synthetic retinoids, induces meflin expression, softens tumor tissues, increases tumor vessel area, and increases intratumoral drug penetration [70, 143]. Clinical trials aimed at testing the efficacy of combination therapy with Am80 and conventional chemotherapy is ongoing [21].

This approach of normalizing CAFs, rather than depleting them, represents a promising strategy in cancer treatment. By targeting the chronic activation of CAFs and reducing excessive ECM production, these methods aim to improve the TME, enhance drug penetration, and promote antitumor immune responses. Drugs like pirfenidone, retinoic acid derivatives, and vitamin D have shown potential in preclinical studies, while the ongoing clinical trials with Am80 highlight the translational potential of this approach. The focus on CAF normalization offers a strategy that may avoid the potential pitfalls associated with complete CAF depletion, possibly leading to more effective and safer cancer therapies.

## Future perspectives in CAF research and targeting

Research on CAFs is advancing, and they are expected to be used in the development of cancer therapies. Below, there are some key areas for further exploration. First, recent advances in single-cell analysis have deepened our understanding of CAF heterogenicity. Future studies should focus on integrating spatial transcriptomics to understand maps showing the spatial distribution of CAFs and the interactions between CAFs and other cells in tumor tissue. Second, research on the utilization of CAFs as markers for predicting prognosis and therapeutic efficiency in cancer treatment and screening is ongoing [144–146]. In addition, profiling individual patients’ CAF landscapes is expected to optimize personalized medicine approaches. Finally, treatment strategies targeting CAFs in combination with conventional therapies may overcome resistance to treatment and improve therapeutic efficacy. Overall, advances in CAF research could lead to the development of more effective cancer treatment strategies that consider the TME.

## Conclusion

I have comprehensively reviewed the heterogeneity of CAFs, including their contributions to therapeutic resistance in cancer. CAFs may be classified into at least five subtypes. Each subtype has a different effect on tumor progression and resistance to therapy. In particular, CAFs reduce the efficacy of conventional cancer therapies through various mechanisms, such as ECM overproduction, immunosuppression, and angiogenesis.

Progress in CAF research has significantly enhanced our understanding of the complexity and dynamics of the TME. In particular, the elucidation of the interactions between CAFs and other cell types, extending beyond ECM production, has revealed mechanisms of treatment resistance and established a foundation for developing new cancer therapies. Thus, CAF research will continue to contribute to the development of cancer therapies.

## Data Availability

Data sharing is not applicable to this article as no datasets were generated or analyzed during the current study.
